# Biological Significance and Targeting of the FGFR Axis in Cancer

**DOI:** 10.3390/cancers13225681

**Published:** 2021-11-13

**Authors:** Athina-Myrto Chioni, Richard P. Grose

**Affiliations:** 1 School of Life Sciences Pharmacy and Chemistry, Kingston University, Penrhyn Road, Kingston upon Thames KT1 2EE, UK; 2 Centre for Tumour Biology, Barts Cancer Institute, Queen Mary University of London, Charterhouse Square, London EC1M 6BQ, UK; r.p.grose@qmul.ac.uk

**Keywords:** fibroblast growth factor, cancer, FGFR inhibitors, FGFR mutations, FGFR signalling, targeting FGFR

## Abstract

**Simple Summary:**

All cells within tissues and organ systems must communicate with each other to ensure they function in a coordinated manner. One form of communication is signalling mediated by small proteins (for example fibroblast growth factors; FGFs) that are secreted by one cell and bind to specialised receptors (for example FGF receptors) on nearby cells. These receptors propagate the signal to the nucleus of the receiving cell, which in turn dictates to the cell how it should react. FGFR signalling is versatile, tightly controlled and important for normal body homeostasis, facilitating growth, healing and replacing old cells. However, cancer cells can take command of this pathway and use it to their advantage. This review will first explain the biology of FGFR signalling and then describe how it can be corrupted, the implications in cancer, and how it can be targeted to improve cancer therapy.

**Abstract:**

The pleiotropic effects of fibroblast growth factors (FGFs), the widespread expression of all seven signalling FGF receptors (FGFRs) throughout the body, and the dramatic phenotypes shown by many FGF/R knockout mice, highlight the diversity, complexity and functional importance of FGFR signalling. The FGF/R axis is critical during normal tissue development, homeostasis and repair. Therefore, it is not surprising that substantial evidence also pinpoints the involvement of aberrant FGFR signalling in disease, including tumourigenesis. FGFR aberrations in cancer include mutations, gene fusions, and amplifications as well as corrupted autocrine/paracrine loops. Indeed, many clinical trials on cancer are focusing on targeting the FGF/FGFR axis, using selective FGFR inhibitors, nonselective FGFR tyrosine kinase inhibitors, ligand traps, and monoclonal antibodies and some have already been approved for the treatment of cancer patients. The heterogeneous tumour microenvironment and complexity of FGFR signalling may be some of the factors responsible for the resistance or poor response to therapy with FGFR axis-directed therapeutic agents. In the present review we will focus on the structure and function of FGF(R)s, their common irregularities in cancer and the therapeutic value of targeting their function in cancer.

## 1. Introduction

Cancer is a disease of cells, starting with genetic alterations in one cell or a small group of cells. If the repair machinery of the cells fails, then accumulation of genetic alterations will lead to cancer and with time to metastasis. In order for cells to become cancerous, they need to adopt behavioural changes outlined as the “hallmarks of cancer” [1]. Of course, besides the classical hallmarks of cancers, many years of research from different angles has shed light onto novel emerging hallmarks of cancer, such as an altered microbiome, neuronal signalling, epigenetic dysregulation and transdifferentiation [2]. There are many targeted therapies that inhibit and block each of the developed competencies necessary for the growth and progression of tumour development. A number of these approaches target tyrosine kinase receptors, such as the human epidermal growth factor receptor 2 (HER2), epidermal growth factor receptor (EGFR), vascular endothelial growth factor receptor 2 (VEGFR2), platelet derived growth factor receptor (PDGFR) and FGFR, in various ways. Acknowledging that many other tyrosine kinase receptors merit therapeutic targeting, this review will focus and discuss further the importance and different ways of targeting the FGF/FGFR axis.

Fibroblast growth factor receptor (FGFR) signalling plays a pivotal role in a myriad of processes including embryonic development, cell differentiation, proliferation, wound healing, cell migration, angiogenesis and various endocrine signalling pathways [3]. Dysregulation of FGFR signalling can lead to an antiapoptotic, mutagenic and angiogenic response in cells, all of which are cancer hallmarks [4]. The oncogenic potential of FGFR signalling also lies in its potential to serve as an escape mechanism for acquired resistance to cancer therapy. To appreciate the therapeutic value of targeting FGFR signalling in cancer, we will first consider normal structure and function, then discuss how aberrant FGFR signalling can influence cancer progression and, finally, describe how it can be targeted.

## 2. FGF(R) Structure

In humans, the fibroblast growth factor (FGF) family comprises 22 members classified into seven subfamilies based on similarities in coding sequence, protein structure and biochemical function: FGF1 (FGF1 and FGF2), FGF4 (FGF4, -5, -6), FGF7 (FGF3, -7, -10, -22), FGF8 (FGF8, -17, -18), FGF9 (FGF9, -16, -20), FGF19 (FGF19, -21, -23) and FGF11 (FGF11, -12, -13, -14) [5,6,7]. Each FGF ligand comprises a conserved core region of 120 amino acids and shares between 35% and 50% sequence homology [6]. Despite being similar in structure, only eighteen FGFs are reported to signal via FGFR, namely FGF1 to FGF10 and FGF16 to FGF23 [8]. Other FGF ligands, such as FGF11 to FGF14, which also share a similar structure to other ligands, do not bind to these receptors but instead can function via voltage-gated sodium channels [9], although recent work casts doubt on this dogma [10]. Five FGF subfamilies (FGF1, -4, -7, -8, -9) are characterised as paracrine signalling molecules that signal by forming a three-way complex with FGFR and heparan sulphate proteoglycans (HSPGs). The other two subfamilies (FGF11 and FGF19) act differently; the FGF11 subfamily act intracellularly, while FGF19 have a reduced HSPG binding affinity and bind to αKlotho and βKlotho cofactors to function in an endocrine manner to have an impact on adult homeostasis and metabolism [8,11].

**Figure 1:**
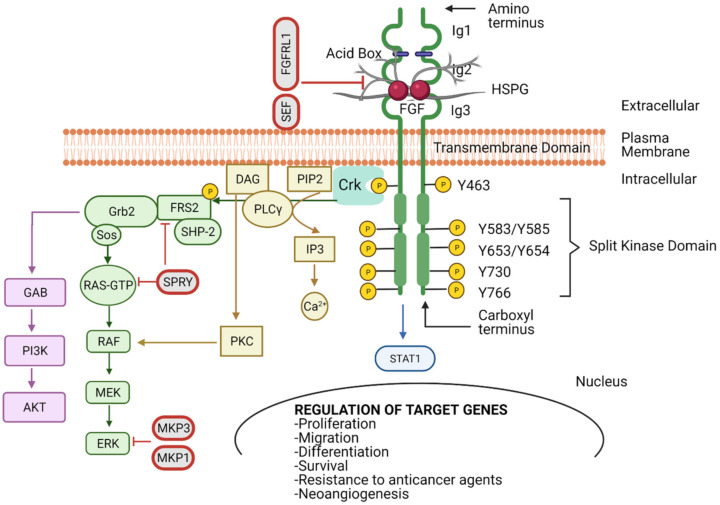
Fibroblast growth factor receptor (FGFR) structure and function. FGFR possesses three immunoglobulin-like domains (Ig1–3), an acid box (AB), a transmembrane domain (TM), and an intracellular tyrosine kinase domain that is split into two (TK1 and TK2). The FGF–FGFR complex is composed by two FGFs, two FGFRs and one heparin sulphate proteoglycan (HSPG). The TK domains are transphosphorylated upon ligand binding between Ig2-Ig3 and receptor dimerisation. This initiates the interaction between a network of downstream signalling molecules that can activate key pathways, such as MAPK, AKT, PLCγ, STAT1 and in turn regulate target genes involved in cell proliferation, migration, differentiation, survival, resistance to anticancer agents and neoangiogenesis. Signalling can be negatively regulated by SEF, FGFR-like 1 (FGFRL1), sprouty (SPRY) and MAPK phosphatase 1 and 3 (MKP1 and MKP3) at different levels. Created with BioRender.com (accessed on 26 September 2021).

**Figure 2:**
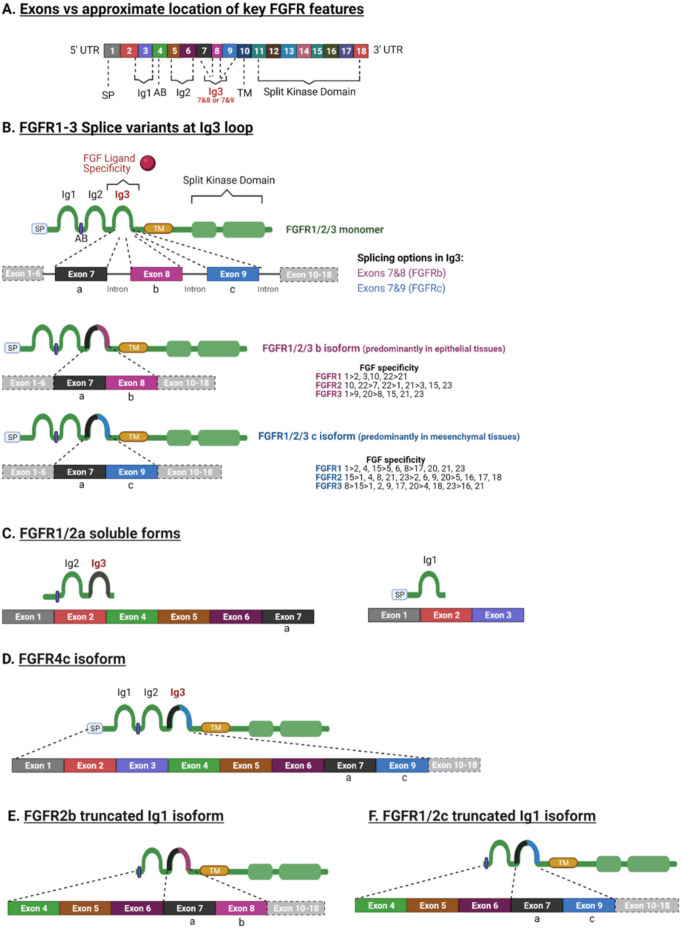
Key FGFR splice variants and ligand specificity. (**A**) A generalised diagram representing the key FGFR features. (**B**) Splice variant at the Ig3 loop occurs in *FGFR1*–3. This splice variant is responsible for ligand binding specificity and is generated by alternative splicing at the Ig3. The first portion of the Ig3 is the exon “a” (exon 7) that is spliced to either exon “b” (exon 8) or exon “c” (exon 9) and then to the exon that encodes the TM domain. The “b” isoform is mainly expressed by epithelial tissues/cells, whereas the “c” isoform is expressed by mesenchymal tissues. FGFs have differential specificity to different isoforms. (**C**) Splice variants can generate soluble variants without TK activity, truncated to one or more Ig domains and missing the TM domain. Variants lacking the TK domain can heterodimerise with full length FGFRs to generate nonfunctional dimers and therefore act as down regulators [24]. (**D**) *FGFR4* can generate a single isoform containing the “c” exon (exon 9) in the Ig3 domain. (**E**) *FGFR2* can generate a splice variant missing Ig1 and Ig3 containing the “b” exon (exon 8). (**F**) *FGFR1* and *FGFR2* can also generate a splice variant with truncated Ig1 and Ig3 containing the “c” exon (exon 9). SP: signal peptide, Ig: Immunoglobulin, AB: acid box; TM: transmembrane domain, UTR: untranslated region. Created with BioRender.com (accessed on 26 September 2021).

FGFRs have extracellular immunoglobulin-like (Ig) domains 1–3 (D1–D3), a transmembrane (TM) domain, tyrosine kinase I, and II domains, a carboxyl-terminal, and an acidic box [12] (Figure 1). The D2 and D3 regions form a ligand-binding pocket for two FGF ligands and two heparin molecules [13]. The acidic box is responsible for the auto-inhibition and regulation of optimal interactions from bivalent cations (Figure 1). The interaction between the acidic box and the heparan sulphate-binding site inhibits activation of the receptor when FGF is absent [8,14,15,16]. FGF binds in the Ig2 and Ig3 domains, where HSPGs protect FGFs from protease-mediated degradation, thus stabilising the FGF–FGFR complex (Figure 1 and Figure 2A) [17]. Hence, high-affinity FGFRs are activated upon FGF ligand binding. Paracrine FGFs bind strongly to HSPGs, which possess cofactor functions to prevent the FGFs from diffusing through the extracellular matrix (ECM) as well as regulating the FGFR specificity [11,18].

Aside from the four main FGFR family members (*FGFR1*–4), there is an additional receptor, fibroblast growth factor receptor like 1 (FGFRL1 or FGFR5), that can bind to FGFs and heparin, but lacks the tyrosine kinase domain and therefore cannot signal via transphosphorylation [19]. *FGFR1*L is believed to negatively regulate FGFR signalling by acting as a decoy receptor that neutralises FGFs by binding to them without activating any downstream signalling cascade [20]. FGFRL1 is expressed mainly in musculoskeletal tissues and the kidney and its main function is to control the growth of the metanephric kidney [21]. It is hypothesised that its function depends on Ig2 and Ig3 domains interacting together with an FGF ligand and another molecule from the surface of other cells from their microenvironment [21]. In fact binding of FGF8 to FGFRL1 plays an important role in developing kidneys by driving the formation of nephrons [22].

## 3. FGFR Splicing

Despite the high sequence homology between FGFR family members (55%–72%) and their similar structural characteristics, there are a variety of isoforms (Figure 2) [23]. FGFR diversity is not only attributed to the different genes that can encode *FGFR1*–4 and the multiple FGFs that can activate them, but also to the fact that FGFR genes can be alternatively spliced (Figure 2).

FGFR genes consists of 18 exons (Figure 2A). Each gene can be alternatively spliced and produce different mRNAs that consequently will result in FGFR protein diversity [25,26]. FGFRs 1–3 each can generate two splice variants of the immunoglobulin (Ig)-like domain 3b and 3c, which are fundamental to ligand-binding specificity during development and are often mis-spliced in cancer [5,8,11]. Hence, there are seven main signalling FGFRs, *FGFR1*b, *FGFR1*c, *FGFR2*b, *FGFR2*c, *FGFR3*b, *FGFR3*c and *FGFR4*, encoded by four genes. Each specific FGFR binds to specific FGFs and most FGF ligands can bind to several different variants of FGFRs. The FGF binding specificity to FGFRs is regulated by two distinct splice variants of exon 8 and exon 9 of domain 3 (D3) [6] (Figure 2A,B). The splicing variant of exon 7/8 and exon 7/9 encodes the carboxyl-terminal of the domain D3, resulting in the -b or -c isoform [27,28,29,30,31]. In human tissues, the -b isoforms are confined to epithelial cells, with the -c isoform predominating in mesenchymal lineages [6]. The specificity of the ligand binding to FGFRs differs amongst isoforms -b and -c (Figure 2B) [6]. For instance, FGF4 binds to the *FGFR1*-3c isoform, while FGF7 binds specifically to the *FGFR1*b and -2b isoforms (Figure 2B). In conclusion, the exon rearrangement at the Ig3 loop (D3) has a profound effect on the FGF spectrum for each receptor, with the *FGFR1*-3b isoforms having a more limited binding affinity with FGFs compared to the *FGFR1*-3c isoforms (Figure 2B) [6].

Interestingly, it has been reported that reversible switching of the *FGFR2*-3b isoform to the -c isoform was induced by exogenous and endogenous FGF1 and FGF2. This switch was confluence and cell cycle dependent [32]. Altered splicing has been associated with cancer progression [33,34,35], for example during EMT [33,36].

*FGFR1* and -2 also have another isoform -a, in which exon 7 joins directly with exon 10, the TM domain. This truncated variant is a secreted protein that is incapable of signal transduction and has an autoinhibitory role [37]. In bladder cancer, the switch from the *FGFR1*a to the *FGFR1*b isoform increased FGF1-induced activation of the latter and was associated with the tumour grade and stage, likely due to it giving a proliferation advantage [38].

In addition, there are secreted FGFR isoforms that lack the TM domain and the entire cytoplasmic region [23,39,40] (Figure 2C). There are also reports of truncated FGFR isoforms lacking Ig1 [39,40,41,42] (Figure 2E). The truncated Ig1 isoform is known to be a high affinity oncogenic variant that can activate various downstream signalling pathways, due to the Ig1 region performing an autoinhibitory role [6,24,43,44]. Interestingly, there are also isoforms missing the acid box between the Ig1 and Ig2 loops (i.e., truncated Ig1 *FGFR2*b and *FGFR3*c) [45] and other isoforms missing the carboxyl terminal [13]. In fact, such a variant missing the inhibitory carboxyl terminal portion of *FGFR2* was expressed in a breast cancer cell line (SUM-52PE), along with other splice variants, with the different splice variants having different transforming activities [43]. Variants expressing the C3 carboxyl terminus resulted in more autonomous signalling, cell growth, and invasion [43]. Recently, a novel *FGFR3* splice variant was reported in African American prostate cancer (*FGFR3*-S) that was associated with a poor prognosis and increased cell proliferation and motility [44]. The *FGFR3*-S variant lacked exon 14, comprising 123 nucleotides that encode the activation loop in the split kinase domain [44]. *FGFR4* is well defined as it is only produced in a single isoform homologous to the *FGFR1*-3c splice variant [46] (Figure 2D). Although their properties are not well understood at present, there are also reports of FGFRL1 isoforms with an absent Ig1 domain with and without the acid box [47].

## 4. FGFR Signalling

The multiple possibilities of FGFR activation, due to the wide range of FGFs and FGFR isoforms, drives several oncogenic signalling pathways. Typically, two FGF molecules are needed to bind to the Ig2 (D2) and Ig3 (D3) extracellular domains in the FGFR to drive dimerisation and activation (Figure 1). Studies on the structure of FGFR revealed that a 2:2 FGF–FGFR complex is formed between the FGF, D2 and D3 of the FGFR. Under physiological conditions FGF–FGFR interactions are not sufficient to stabilise FGFR dimers [14], with HSPG acting as a linker to stabilise the HSPG–FGF–FGFR complex [6,36,48,49]. The FGF–FGFR–HSPG complexes induce activation of downstream signalling cascades; mitogen-activated protein kinase (MAPK), phosphatidylinositol 3-kinase (PI3K), phospholipase Cγ (PLCγ) and signal tranducer and activator of transcription 1 (STAT 1) (Figure 1) [50].

As discussed, the specificity of FGF–FGFR binding is determined by alternative splicing, ligand specificity and tissue specific expression of both FGF ligands and receptors [5,6]. Further control of the FGF–FGFR coupling is provided by the interaction with secreted proteins and plasma membrane bound receptors, such as α and β Klotho proteins [5,51,52], and a single-pass transmembrane Klotho-related protein (KLPH). These act as cofactors for the endocrine FGFs by forming an FGF–FGFR–Klotho ternary complex [53,54].

Dimerisation of the FGFR causes a conformational shift in the receptor’s structure, leading to a 50- to 100-fold increase in the receptor kinase activity, resulting in the phosphorylation through mutual transphosphorylation of numerous tyrosine residues in the intracellular domain. Subsequently, various protein complexes are formed to initiate downstream signalling transduction [4,9,11,12,55,56]. One of the adaptor proteins that governs the downstream signalling cascade is the v-crk sarcoma virus CT10 oncogene homolog (Crk) (Figure 1) [48]. Upon FGFR phosphorylation, Crk gets transiently phosphorylated and can be associated with son of sevenless (SOS), that in turns activates small GTPases [49,51,52,57].

Furthermore, FGFR activation can trigger the phosphorylation of the docking protein FGFR substrate 2 (FRS2), accompanied by the recruitment of shp2 tyrosine phosphatase. Phosphorylation of shp2 facilities its link with growth factor receptor-bound 2 (GRB2) and SOS [55,56,58,59,60]. The recruitment of GRB2 associated binding protein 1 (GAB1) forming the FRS2 complex leads to the activation of the PI3K-Ak strain transforming (AKT) pathway, which regulates cell survival and fate [61,62,63] (Figure 1). Several other signalling molecules have also been reported to be activated by FGFRs, STATs, p90 ribosomal protein S6 kinase 2 (RSK2) and the nonreceptor tyrosine kinase Src [64,65,66,67]. The phospho-tyrosine residues in FGFR carboxyl terminal regions confer strong and selective binding to src homology two (SH2) domains accommodating proteins such as PLCγ. These interactions result in phosphatidylinositol 4, 5-bisphosphate (PIP2) hydrolysis to produce inositol-1,4,5-trisphosphate (IP3) and diacylglycerol (DAG) [68,69,70]. IP3 accumulation stimulates Ca^2+^ release from internal stores, while activation of protein kinase C (PKC) and MAPK pathways are facilitated by DAG [71] (Figure 1).

With the inherent complexity of the modes of activation, transduction and biological output, it is not surprising that the orchestration of FGFR signalling is tightly regulated. We previously discussed the FGFR autoregulation via the acid box in Ig1 and its association with ligand binding Ig2 and Ig3 domains (Figure 1). However, there are several mediators associated with controlling the signalling output from activated FGFRs. Some of the known negative regulators are sprouty proteins that are induced by FGF signalling (Figure 1) [72,73]. Furthermore, FRS2 can be phosphorylated by MAPK on serine and threonine residues, inhibiting GRB2 recruitment and producing a negative feedback loop [74,75]. Other negative modulators of the FGF signalling pathway are the transmembrane proteins, similar expression to FGF genes (SEF), anosmin-1, fibronectin-leucine-rich transmembrane protein 3 (FLRT3), FGFRL1 and MAPK phosphatases (MKP) that can also interfere with the activation of downstream signalling pathways [76,77,78,79] (Figure 1). In addition to the above, the stimulated FGF–FGFR complex can be completely blocked by internalisation and subsequent lysosomal degradation. The E3 ubiquitin ligase Cbl binds to activated FRS2 and facilitates FGFR ubiquitination by acting as a signal for receptor degradation [80].

FGFR signalling clearly has profound direct effects on cancer cells. However, the FGFR axis also impacts on angiogenesis and this is an emerging field of translational medicine [81]. FGF2 has been heavily implicated as a proangiogenic factor, promoting endothelial proliferation and migration following FGFR1/2 signalling and VEGF/angiopoietin 2 secretion [82], and has been shown to mediate resistance to VEGFR targeted therapy in cancer [83]. In addition, other FGFs, such as FGF5 and FGF18, can promote angiogenesis through endothelial FGFR activation [84,85]. Interestingly, a recent study revealed an association of an *FGFR1* mutation with spontaneous haemorrhage in paediatric and young adult low grade glioma, though the specific mechanism remains unclear [86]. In urothelial carcinomas, *FGFR3* was able to induce a proangiogenic phenotype, suggesting that constitutive activation of *FGFR3* may be able to potentiate growth factor signalling in the tumour microenvironment and implicating *FGFR3* as a potential therapeutic target from an antiangiogenic perspective [87]. As with other behaviours, the effects of FGFR signalling can be context specific. In an embryoid body model, *FGFR1* negatively regulated angiogenesis by altering the balance of cytokines, such as interleukin-4 and pleiotrophin [88].

## 5. Examples of the Involvement of FGFR Signalling in Development

Before discussing how FGFR signalling can drive cancer, it is important to understand how it is involved in development and why such a pleiotropic and dynamic pathway can be key in disease development. FGFR signalling plays a fundamental role in cell proliferation and migration. However, during embryonic development, FGF signalling regulates differentiation and the cell cycle. FGF signalling is important as early as in the preimplantation of embryos in mammals. For example, FGF4 is expressed in the morula and then in epiblast cells of the inner cell mass [89] and facilitates cell proliferation and the formation of the ectoderm [90,91]. There are reports of *FGFR1* and *FGFR2* in the inner cell mass and *FGFR2* also in the embryonic ectoderm [92]. Later in development it has a vital role in organogenesis, particularly regulating the reciprocal crosstalk between epithelial and mesenchymal cells [93,94]. For example, *FGFR2* plays an important function in both the ectoderm and mesenchyme during limb development [7]. More broadly, mesenchymal cells express FGFs, such as FGF4, 7, and 10, leading to downstream signalling activation through the epithelial 3b splice variant of *FGFR1* and -2 in the epithelium and as a result, facilitate lung, salivary gland, intestine and limb development [95,96,97]. In contrast, epithelial tissue can secret FGFs 8 and 9 that activate *FGFR1* and *FGFR2*-3c isoforms in the mesenchymal tissue [98,99]. However, organogenesis is not always driven exclusively via paracrine loops [11]. During development of the central nervous system, FGF8 signals in the anterior neural primordium by acting as an autocrine/paracrine factor in the development of the inner ear [100]. The differentiation of the cochlear sensory epithelium is regulated by autocrine/paracrine FGF signalling [101]. More recently it was found that FGFR can interact with N-Cadherin and activated FGFR that in turn facilitates migration of neocortical projection neurons [102]. FGFRs could regulate multipolar neuronal orientation and change them into bipolar cells to enter the cortical plate [102].

Given the importance of FGF signalling to development, it is unsurprising that malfunction can lead to developmental defects. The absence of *FGFR1* in genetically modified mice leads to early growth defects [103]. Activated FGFR germline mutations can lead to skeletal disorders, such as a mutation in *FGFR3* which can lead to growth defects and human dwarfism achondroplasia (ACH) [104,105]. A variety of inherited syndromes are caused by germline irregularities in FGFR [106]. Furthermore, mutations, especially in *FGFR2*, can lead to craniofacial malformation syndromes [107].

## 6. Aberrant FGFR Signalling in Cancer

**Figure 3:**
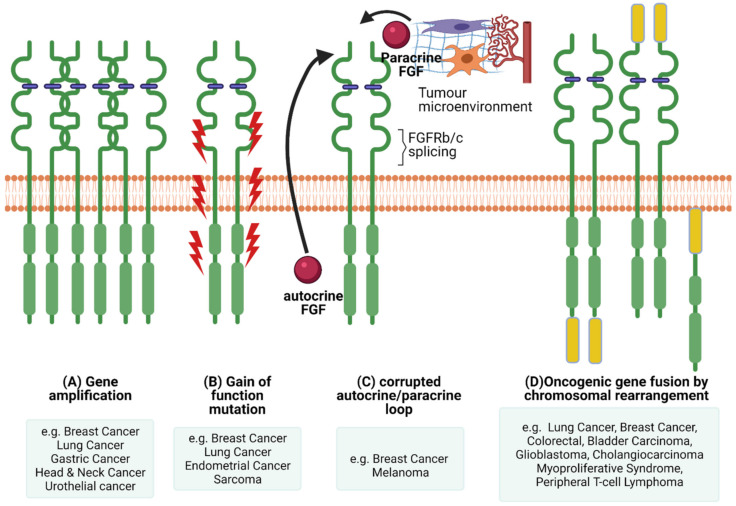
FGFR dysregulations. There are several mechanisms of oncogenic FGFR function. (**A**) Gene amplification results in accumulation of FGFRs that usually translate into protein overexpression and activation of the FGFR axis. (**B**) Gain of function mutations can lead to constitutive FGFR activation with or without FGF binding. (**C**) Corrupted autocrine and paracrine loops, either via alternative splicing affecting ligand binding specificity or FGFRs getting overstimulated by FGFs produced in an autocrine fashion, by the cancer cells themselves, or by the tumour microenvironment, in a paracrine fashion. (**D**) Chromosomal rearrangements can lead to the creation of hybrid oncogenic FGFRs by fusing with binding partners at the carboxyl or amino termini. Created with BioRender.com (accessed on 7 November 2021).

**Figure 4:**
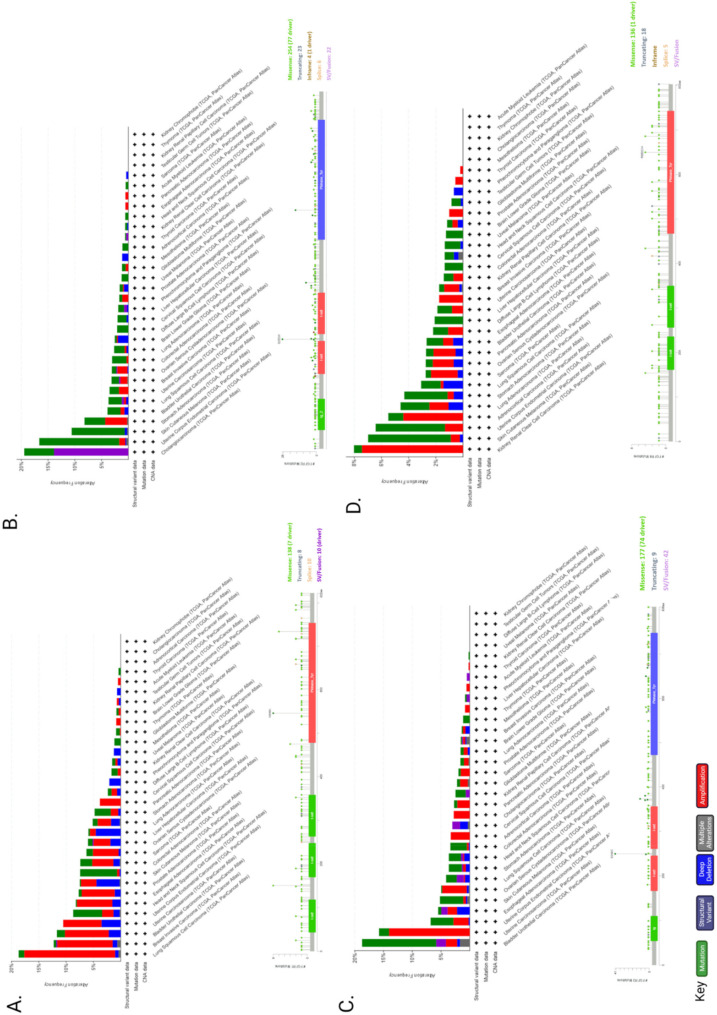
*FGFR1*–4 genetic alteration in cancer. Genetic alterations by cancer type and mutations of *FGFR1* (**A**), *FGFR2* (**B**), *FGFR3* (**C**) and *FGFR4* (**D**) were found in 662 (~6%), 360 (~3%), 351 (~3%) and 285 (~3%) patients, respectively out of 10,953 in total using cBioPortal. Mutation diagram circles and histograms are coloured with respect to the corresponding mutation types: Green = gene mutations; purple = structural variants; blue = deep deletions; grey = multiple alterations; red = gene amplifications. The lollipop diagrams of each FGF receptor (*FGFR1*–4), below each histogram, represent the mutation types in relation to the gene location (i.e., missense, truncating, in-frame, splice, SV/fusion). In the case of different mutation types at a single position, the colour of the circle is determined with respect to the most frequent mutation type.

The pleiotropic function of FGFR and its involvement in crucial physiological processes makes the FGFR signalling pathway a good candidate for facilitating cancer progression. In this section we will highlight the different ways FGFR signalling can be involved in the pathogenesis of cancer (Figure 3) and briefly give examples of FGFRs’ genetic alterations in different cancers (Figure 4).

One way of facilitating malignant progression via FGFR signalling is via a corrupted autocrine/paracrine loop and exon switching. Dysregulation of FGF secretion and FGFR expression in stromal and cancer cells can be a driving force in cancer progression. Many FGFs and their elevated levels are associated with cancer progression, for example FGF1, -2, -6, -8, -10, -19 and -23 [108,109,110,111,112,113,114,115,116,117,118]. Interestingly, FGFs are implicated in EMT in cancer by attributing mesenchymal characteristics in epithelial cells [119,120,121]. In some cases, growth factors (e.g., FGFs) are produced and secreted by one type of cell (for example stromal cells) and stimulate via paracrine signalling another type of cell to signal cell functions, such as proliferation, differentiation and migration [1].

However, cancer cells can synthesise FGFs and create a positive feedback loop via autocrine signalling. For example, in breast and non-small cell lung carcinomas, FGF2 and FGF9 are expressed and activate their respective FGFRs in the same cells [122,123]. Furthermore, FGF10 has been implicated as a key paracrine signal in breast, pancreatic, stomach, skin, lung and prostate cancer [108,124].

The specificity of FGF ligands can be altered through isoform switching and alternative splicing of FGFRs, thereby increasing the range of FGFs that can stimulate cancer cells, depending on the FGFR isoforms they express [8,35]. For example, alternative splicing of *FGFR1* is associated with a high tumour grade and stage in bladder cancer [38]. Similarly, *FGFR1* alternative *FGFR1*α/*FGFR1*β splicing was found to play a key role in breast cancer [34] and *FGFR3* splicing promoted aggressiveness in prostate cancer [125].

Deregulation of negative regulators of the FGFR axis can also contribute to aberrant FGFR signalling in cancer. For example, SEF and SPRY expression levels are associated with breast, prostate, ovarian and thyroid cancer progression, with high grade carcinomas expressing lower levels of these negative FGFR regulators [126,127,128]. In contrast, a recent study reported that loss of SPRY1 improved the response to targeted therapy in melanoma [129] and suppression of SPRY1 inhibited triple-negative breast cancer malignancy via enhancing the estrogen growth factor and its receptor (EGF/EGFR) mediated mesenchymal phenotype [130].

Genetic alterations of FGFR can also dysregulate signalling and contribute towards malignant progression. Next-Generation Sequencing (NGS) analysis of 4853 tumours revealed FGFR aberrations in 7.1% of cancers [131]. More specifically, 66% of the aberrations were gene amplification, 26% were gene mutations and 8% were rearrangements [131]. A recent study on advanced urothelial cancer using NGS to analyze cell-free DNA from the plasma of 997 patients, revealed that 20% had *FGFR2* and *FGFR3* genomic alterations, of which 14% were activating mutations [132].

### 6.1. Activating Mutations

The most common types of genetic variation are single nucleotide polymorphisms (SNPs). *FGFR2* harbours one of the first SNPs to be identified as a breast cancer susceptibility locus by Genome-Wide association studies (GWAS) [133,134]. Risk alleles of various SNPs found in *FGFR2,* they are associated with ER-positive cancers [135], increased FGFR2 expression [136], lymph node metastasis in breast cancer [137] and radiation-induced breast cancer risk [138]. More recently another study identified an *FGFR2* SNP that was linked with susceptibility to breast cancer in a Chinese population [139]. However, only a few SNP loci are confirmed in *FGFR1* that correlate significantly with a breast cancer predisposition [140]. In contrast, a more recent study correlated three *FGFR1* SNPs to reduced breast cancer risk [141]. SNPs in *FGFR4* but not in *FGFR3* were strongly correlated with breast cancer [142]. In breast cancer patients, *FGFR4* and *FGFR2* SNPs were previously suggested to be candidate pharmacogenomic factors to predict the response to chemotherapy [143]. Notably, SNPs in the FGF/FGFR axis (FGF1, FG18, FGF7, FGF23 and FGF5) were also associated with an increased risk of ovarian cancer [144].

A number of germ line activating point mutations in *FGFR1*, -2 and -3 are found in prostate, breast, bladder, endometrial, brain, lung, uterus, cervical, stomach, head and neck, colon and melanoma cancers (as reviewed by [145]). These mutations can alter the ligand binding, juxtamembrane and kinase domains and constitutively activate FGFR or impair FGFR degradation, leading to increased FGFR signalling (as reviewed by [111,145,146,147]. *FGFR4* activating mutations are not detected very often, apart from in rhabdomyosarcoma [148] and gastric cancer [149]. Interestingly, some of the activating mutations can result in changes in the efficacy of several inhibitors that can target FGFR, such as AZD4547, BGJ-398, KTI258, AP24534 and JNJ42756493 [150].

### 6.2. FGFR Gene Amplification and Overexpression

Elevated FGFR levels can be achieved either via chromosomal amplification or aberrant transcription (Figure 3). In cancer, distinctive FGFR abnormalities are known such as the amplification of genes or post-transcriptional regulation, contributing to overexpression of the receptor. Mutations in FGFRs could generate receptors that are either consistently active or may demonstrate a reduced necessity of activation through ligand binding [11]. The most common abnormalities in malignancies are due to gene amplification of *FGFR*1, -2 and -3, as well as FGF ligands. Several studies have highlighted that *FGFR* is amplified in various cancers. For example, *FGFR1* expression is amplified in bladder, oral, oesophageal squamous, NSCLC, prostate and ovarian cancers [151,152,153,154]. *FGFR1* amplification and overexpression was observed in some patients with lymph node metastasis and advanced pathological stages of hypopharyngeal and laryngeal squamous cell carcinoma [155]. In addition, hormone receptor positive breast cancer patients with metastatic disease had *FGFR1* amplification that was associated with a shorter time to progression on first line endocrine therapy [156]. Furthermore, it was suggested that *FGFR1* amplification grants resistance to estrogen receptor (ER), PI3K, mammalian target of rapamycin (mTOR) and cyclin-dependent kinase (CDK)4/6 inhibitors [157]. *FGFR2* amplification in gastric cancer is associated with a poor prognosis and response to chemotherapy [158].

### 6.3. Chromosomal Translocation

The exchange of chromosomal arms (or segments) between heterologous chromosomes, known as chromosomal translocation, is a type of structural chromosomal abnormality that results in fusion genes/proteins. The generated fusion proteins can have oncogenic properties. Chromosomal translocations in FGFRs have about an 8% incidence rate [131]. There are two types of *FGFR* gene fusions: (1) when only the FGFR tyrosine kinase domain is fused to the 5′ end of the fusion protein (the extracellular and transmembrane domain portion of the FGFR is missing from the fusion protein), therefore is constitutively dimerised and active; (2) when the whole FGFR remains intact and acts as the 5′ fusion gene that will bind to its partner at the 3′ end of the FGFR [147].

The first reports of FGFR fusion genes were in haematological malignancies. The FGFR kinase domain was fused with the N terminus of transcription factors such as ETV6, ZNF198 and BCR in lymphoma/leukaemia patients with myeloproliferative disorder stem cell syndrome [159,160,161,162]. A recent study reported *EVT6-FGFR2* fusion protein in a mixed phenotype (T-myeloid/lymphoid) acute leukaemia, that resulted in aberrant *FGFR2* tyrosine kinase expression and was correlated with aggressive clinical behaviour and a poor response to therapy [163]. *FGFR1*, *FGFR2* and *FGFR3* fusions are also identified in solid tumours, such as lung, colorectal, glioblastoma, breast, head and neck, bladder, cervical cancer and cholangiocarcinoma (as reviewed by [164]). A common fusion is *FGFR3* with transforming acidic coiled-coil 3 (TACC3) that induces a constitutive phosphorylation of the tyrosine kinase domain and therefore activation of downstream MAPK and STAT1 pathways that further leads to increased metastatic cell behaviour (e.g., cell proliferation) [165,166,167]. There are several identified binding partners for *FGFR2*, some of them are TACC3 and CCDC6 in cholangiocarcinoma [166,168] and BICC1 in hepatocarcinoma and colorectal cancer [169]. Examples of *FGFR1* fusion partners are HOOK3 in gastrointestinal stromal tumour, TACC1 in glioblastoma and ZNF703 in breast cancer [167,170,171,172]. A recent genomic profiling study identified ANO3 and NSD1 as fusion partners for *FGFR4* in non-small cell lung cancer [173]. Although FGFR fusions are relatively rare in human cancers it might be of interest to identify how patients with FGFR fusions respond to therapy targeting the tyrosine kinase (TK) domain of FGFR.

## 7. Nuclear FGFR in Cancer

FGFRs have been shown to signal via the cell membrane and endosomal compartments via downstream signalling pathways. However, studies have suggested that other TK receptors as well as FGFRs and FGFs, can target the nucleus and carry out functions that might not be dependent on tyrosine kinase activity [174,175,176,177,178,179,180,181,182]. Examples of nuclear FGFs are FGF1, that stimulated DNA synthesis, and FGF2 that was associated with increased cell proliferation in glioma cells and invasion in pancreatic cancer [11,183,184]. Both *FGFR1* and *FGFR2* have been reported to function in the nucleus [183,185,186]. Nuclear *FGFR2* was recently found to negatively regulate hypoxia-induced cell invasion in prostate cancer [187] and nuclear *FGFR1* was positively corelated with pancreatic and breast cancer progression [178,179].

Although there are strong indications, it is still not fully understood how FGF(R)s travel to the nucleus and what their mode of action is once there. Several researchers have highlighted the mechanisms by which full length TK receptors translocate via the cell membrane to the nucleus. For example, upon binding of the ligands, the activated receptors get internalised to the early endosomal compartments either via the vesicular pathway or after retro-translocation from the endoplasmic reticulum (ER) to the cytosol [181,188,189,190]. The molecular mechanism by which the receptor escapes the endosomal pathway to travel to the nucleus remains elusive and conflicting data point to different trafficking possibilities. One of the possible mechanisms for nuclear translocation of full length FGFR involves retro-translocation of FGFR from the ER/Golgi apparatus [183]. Typically, after co-translational insertion into the ER membranes, *FGFR1* traffics via the vesicular transport systems through the Golgi apparatus to reach the plasma membrane [185,191]. This process may be accompanied by retro-translocation of the pool of FGFR into the cytosol, with *FGFR1* undergoing retrograde transport via the sec61 channel, similarly to ER-associated protein degradation [183]. Once in the cytosol, FGFRs interact with ribosomal S6-kinase 1 and FGF2 which facilitates receptor transport to the nucleus to directly regulate gene expression [185,191]. Full length FGFR is a molecule too large to pass through the nuclear membrane via diffusion, and another mechanism involves the full-length receptor in the cytoplasm activating the importin beta pathway to enter the nucleus [176]. The nuclear receptor can then interact with other nuclear proteins to control transcription [185,192,193].

An alternative is that the nuclear trafficking of the receptor is dependent on proteolytic cleavage of the intracellular domain allowing translocation to the nucleus of the unrestricted cytoplasmic portion [179,194]. There are several mechanisms utilised by tyrosine kinase receptors to reach the nucleus, but generation of nuclear RTK fragments via alternative splicing of the receptor or proteolytic cleavage of FGFRs/RTK with caspases, secretases, granzymes and other proteases (e.g., ADAM10/15/17) [179,184,186,188], are increasingly reported. The FGF receptor can be present in a cleaved form before trafficking to the nucleus, and there are indications suggesting this proteolytic pathway might be FGFR kinase activity-dependent [179]. Previous studies indicated that Notch1 and *FGFR1* can be cleaved by Granzyme B (GrB) [189]. In breast cancer cells, FGFR activation-dependent cleavage of *FGFR1* generates a C-terminal fragment that can translocate to the nucleus and control the expression of target genes [179]. Nuclear *FGFR1* could control the oncogenic networks involved in organ development, tissue and cell pluripotency, cell cycle, cancer related *TP53* pathway, neuroectodermal and mesodermal programming networks, axonal growth and synaptic plasticity pathways [190].

Therefore, there might be a novel mechanism by which FGFR signalling can control metastatic cancer cell behaviour. This further suggests a potentially novel therapeutic target for invasive cancer treatment.

## 8. Targeting FGFR Signalling in Cancer

One of the main obstacles in cancer therapy is chemoresistance and radioresistance. There is evidence highlighting the possible role of the FGFR axis in the development of drug resistance. For example, overexpression of FGF2 and FGF1 are linked with both in vivo and in vitro resistance to cancer drugs such as doxorubicin, 5-fluorouracil and paclitaxel [195]. Interestingly, a pan-FGFR inhibitor (BGJ398) was able to overcome paclitaxel resistance in FGFR1 expressing urothelial carcinoma [192]. Another study identified *FGFR4* as a targetable element of drug resistance in colorectal cancer [193]. Increased *FGFR1* and FGF3 expression was correlated with a poor response to anti-HER2 treatment in breast cancer patients, and this was overcome using a combination therapy of FGFR inhibitors together with lapatinib and trastuzumab [196]. Overexpression of *FGFR3* was also linked with tamoxifen resistant breast cancer [197]. In afatinib-resistant non-small cell lung cancer cells, overexpression of *FGFR1* and FGF2 played a role in overcoming cell survival by compensating the loss of the estrogen growth factor receptor (EGFR)-driven signalling pathway [198]. In addition, gefitinib sensitivity was also restored in non-small cell lung cancer cells when FGF2 and *FGFR1* were inhibited via siRNA and treatment with a small molecule inhibitor, PD173074, suggesting FGFR activation as a potential mechanism of acquired resistance to EGFR-TKs [199]. In *FGFR1* amplified lung cancer, a combination therapy approach overcame resistance to treatment with an FGFR inhibitor [200]. In EGFR-dependent cancers of multiple cell lineages, *FGFR3*-TACC3 fusion proteins are also characterised as “naturally occurring drivers of tumour resistance” by reactivating EGFR/ERK signalling [201]. Considering all the evidence together, this highlights the importance of targeting the FGFR axis in combination therapies tailored for different cohorts of patients.

**Figure 5:**
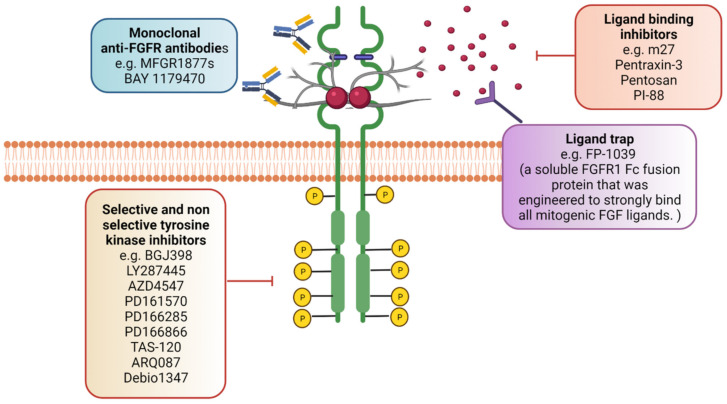
Targeting the FGFR axis. Aberrant FGFR signalling can contribute to cancer progression and therefore targeting FP-1039. can also block the FGF–FGFR interaction and therefore prohibit FGFR activation. In addition, ligand binding inhibitors that act as antagonists (e.g., PI-88 and sm27) can prevent FGFR activation. Monoclonal antibodies targeting specific FGFR isoforms (e.g., MFGR1877s by Genentech) can also have anti-tumour activity. Created with BioRender.com (accessed on 12 November 2021).

Therapeutic targeting of FGFs and their receptors is a key area of drug development. Several drugs targeting FGF pathways are currently under clinical investigation (Table 1). However, abrogating FGFR signalling can be accomplished by targeting the diverse components present in the pathway, which include the ligands, receptors as well as the products of the downstream signalling pathway [61] (Figure 5). Nevertheless, converting knowledge into a treatment for patients has proven challenging as even specific inhibitors targeting FGFR have off-target effects [202,203,204]. Hence, further research is necessary to determine the mechanisms of effective targeting of FGFR signalling in cancer without obstructing its fundamental functions in healthy cells.

The FGFR targeting field has progressed significantly, as novel agents inhibiting FGF ligands or using monoclonal antibodies and FGF ligand traps have been developed as well as using FGFR non-selective and selective inhibitors (Figure 4). The ATP-competitive small molecules were the first FGFR inhibitors [205,206]. PDGFR and VEGFR share comparable structural homology to FGFRs, hence these inhibitors can act as multitarget tyrosine kinase inhibitors (TKIs) as they also bind and act on the conserved ATP-binding regions.

One of the non-selective FGFR TKIs is dovitinib (TKI 258, Novartis, Basel, Switzerland), which is in phase II/III clinical trials, and this has been shown to have a strong affinity to *FGFR3* resulting in the inhibition of downstream signalling, blocking cell proliferation and promoting apoptosis [61,207]. Dovitinib likewise inhibits other members of the TK family due to a lack of drug specificity including *FGFR1*, PDGFR and VDGFR [208,209,210]. A pilot study evaluated the efficacy of an orally bioavailable multitargeted tyrosine kinase inhibitor, ponatinib, that inhibits all FGFRs as well as other kinases (such as KIT, RET, SRC, VEGFR and PDGFR) [211,212]. Their findings demonstrated a clinical benefit response in over 45% of the patients, suggesting a potential antitumour activity of ponatinib in biliary tract cancer patients with altered *FGFR2* [212]. AZD4547 (AstraZeneca, Cambridge, UK) is a highly potent selective *FGFR1*-3 inhibitor. Phase I/II clinical trials have indicated that AZD4547 can target cancers, such as gastric/esophagogastric, bladder, gastric adenocarcinoma, lung and breast, with *FGFR1* and -2 amplifications [213,214,215,216,217,218,219,220]. A recent detailed literature review using a wide range of databases and utilising a systematic review approach, demonstrated that clinical trials using selective FGFR inhibitors (i.e., erdafitinib JNJ 42756493, Infigratinib BGJ398, Rogaritinib BAY 1163877, PD173074, BLU9931, AZD4547, Pemigatinib INCB54828, LY2874455, DEBIO 1347, Futibatinib TAS-120) in advanced urothelial cancer had significant antitumour activity [221].

Infigratinib (a pan-FGFR kinase inhibitor) was evaluated in a phase 2 study for biliary tract carcinoma with FGFR alterations, with all responsive tumours containing *FGFR2* fusions. The overall response rate for *FGFR2* fusions was 18.8% and the disease control rate was 83.3% with an estimated median progression-free survival of 5.8 months [222]. Currently there are seven phase 1 and 2 clinical trials evaluating Infigratinib in gastric, adenocarcinoma, breast, advanced malignant solid neoplasm, bladder, renal pelvis and ureter urothelial carcinoma, advanced cholangiocarcinoma and glioblastoma (NCT05019794, NCT04504331, NCT04233567, NCT04972253, NCT04197986, NCT04228042, NCT02150967, NCT04424966). Most importantly, there are two phase 3 clinical trials investigating Infigratinib as a possible cancer treatment for upper tract urothelial carcinoma/urothelial bladder cancer (NCT04197986) and advanced cholangiocarcinoma (NCT03773302).

In trials using Erdafitinib (another a pan-FGFR kinase inhibitor), the rate of confirmed response in advanced metastatic urothelial carcinoma was 40%, with the median duration of progression-free survival at 5.5 months, and median duration of overall survival at 13.8 months [223]. In a phase 2 study on cholangiocarcinoma patients with FGFR alterations, it was reported that the disease control rate was 83.3% and median progression free survival was 5.59 months. In 10 evaluable *FGFR2*+ patients the disease control rate was 100% and the median progression-free survival was 12.35 months [224]. Currently, there are nineteen phase 1 and 2 clinical trials on Erdafitinib and cancers such as breast, bladder/urinary bladder, lung, advanced solid tumours, urothelial, prostate, and multiple myeloma (NCT03238196, NCT04917809, NCT04172675, NCT02699606, NCT04083976, NCT02365597, NCT03547037, NCT03999515, NCT04754425, NCT05052372, NCT03827850, NCT03210714, NCT03473743, NCT04963153, NCT03955913, NCT03088059 NCT02925234, NCT02465060, NCT03732703, NCT03155620). There is also a phase 3 clinical trial evaluating Erdafitinib in urothelial cancer (NCT03390504).

Another pan-FGFR inhibitor, Rogaratinib, showed excellent in vivo efficacy in FGFR overexpressing preclinical cancer models [225]. There are five phase 1 and 3 clinical trials studying Rogaratinib in breast, lung, gastrointestinal stromal, urothelial, and squamous cell head and neck cancers (NCT04483505, NCT03762122, NCT04595747, NCT03473756, NCT03088059).

Therapeutic monoclonal antibodies have been established with the rationale that they could target FGF ligands and FGFR isoforms with a high specificity, hence offering an alternative to inhibitors that might have side effects [226,227]. Antibodies can compromise the other benefit of employing the immune system to synergise with the antitumour activity via antibody-dependent cellular cytotoxicity or complement-dependent cytotoxicity. A number of anti-FGFR monoclonal antibodies have also been considered in preclinical studies [228,229]. Human anti-*FGFR3* mAb, MFGR1877S (Genentech), is a monoclonal antibody against *FGFR3* and has been used against multiple myeloma and MFGR1877S and has also shown antitumour activity for overexpressed *FGFR3* in preclinical models of bladder cancer [221,229,230,231,232,233]. Phase I clinical trials of MFGR187S have been carried out in t(4;14) translocated multiple myeloma patients [233]. Furthermore, GP369 is a specific and potent anti-*FGFR2*b monoclonal antibody that suppresses phosphorylation and the downstream signalling induced by ligand binding. *FGFR2* activated signalling in mice significantly inhibited the growth of human cancer xenografts in the presence of GP369 [234,235].

Antibodies against FGF2 and FGF8 have also shown promising results in inhibiting tumour progression and angiogenesis [236,237] A human single-chain variable fragment (ScFvs; 1A2) that binds to human FGF2 was identified via screening of a human scFv phage library [238]. This purified antibody inhibited various biological functions of FGF2, such as proliferation/growth, migration and tube formation of human umbilical vein endothelial cells and apoptosis in glioma cells in vitro [238].

An alternative method of inhibiting FGFR signalling is via a ligand trap to isolate FGF ligands preventing them from binding to and activating FGFRs [112,239,240]. FP-1039 (GlaxoSmithKline, GSK3052230) is a soluble fusion protein that consists of an extracellular *FGFR1*-IIIc domain fused to the Fc portion of IgG1 that inhibits the binding of FGF1, -2, and -4 to *FGFR1* and has shown promising results in solid tumours [241,242,243]. Other FGF2 antagonists are small molecules such as sm27, PI-88, pentosan and pentraxin-3 [8]. Because of the ability to bind to heparin/heparan sulphate, chemical compounds mimicking heparin (i.e., suramin) could antagonise FGF2 binding and inhibit its action [244]. Peg-interferon alpha-2b was also able to suppress the plasma FGF2 level in melanoma patients with metastasis and gave a clinical response [245]. FGF2-induced angiogenesis was also inhibited by sulfonic acid polymers such as PAMPS, small molecules such as sirolimus, PI-88, thalidomide, suramin and platelet factor 4 protein (as reviewed by [246]).

Not much is known about the mechanism by which FGFR inhibitors induce cell death. Recent work on endometrial cancer showed that FGFR inhibitors (Infigratinib, AZD4547 and PD173074) caused mitochondrial depolarisation, cytochrome c release and impaired mitochondrial respiration in two *FGFR2*-mutant endometrial cancer cell lines (AN3CA and JHUEM2). However, they did not detect caspase activation following FGFR inhibition. When they were treated with the pan-caspase inhibitor (Z-VAD-FMK) they did not prevent cell death, suggesting that the cell death was caspase-independent [247]. Bcl-2 inhibitors enhanced FGFR inhibitor-induced mitochondrial-dependent endometrial cancer cell death [247]. Interestingly, in another study, Infigratinib induced cell death in non-small cell lung cancer cells (H1581) by activating the caspase-dependent mitochondrial and non-mitochondrial pathway [239]. In high-grade bladder cancer cells, a combination treatment with Infigratinib and a novel histone deacetylase inhibitor (OBP-801/YM753/spiruchostatin A), inhibited cell growth and markedly induced apoptosis, by activating caspase-3, -8 and -9. Interestingly, a pan-caspase inhibitor (Z-VAD-FMK) significantly reduced the apoptotic response to the combined treatment. The combination treatment was shown to be at least partially dependent on Bim [240].

## 9. Conclusions

Even though drugs targeting tyrosine kinase activity (e.g., HER2, FGFR, EGFR, VEGFR2), can prolong survival by inducing cancer regression, the lack of selectivity to a single target and/or development of drug resistance remains a problem. The heterogeneous nature of cancer, the involvement of the tumour microenvironment, together with the pleiotropic way FGFR signalling functions, highlights the need for a more personalised approach in cancer treatment and combination therapies. Experimental data and clinical trials focusing on targeting the FGFR axis have demonstrated positive outcomes. An awareness of FGFR genetic alterations or the FGFR mode of action in cancer patients (e.g., whether FGFR acts via a paracrine or autocrine mechanism in a specific tumour) is important for tailoring combinations of targeted therapies aiming at the FGFR axis. For example, using small molecule FGFR inhibitors, RNA based drugs, FGF traps and humanised/human anti-FGFR monoclonal antibodies in combination with targeting the immune system and/or other signalling pathways. A better understanding of FGFR biology could also help in identifying the mechanisms of drug resistance to FGFR inhibitors and facilitating their bypass. Developing diagnostic assays to screen patients for FGF and FGFR status for a targeted approach might help improve treatment efficacy.

## Figures and Tables

**Table 1:** Phase 3 Interventional clinical studies targeting FGF receptors in cancer. Currently there are no phase 4 clinical trials, however, there are over 90 phase 1 and 2 clinical trials targeting FGFR in different types of cancers, and a number of phase 3 trials not yet recruiting. FGFR inhibitors are indicated in bold.

NCT Number	Title	Conditions	Interventions	Enrolment
NCT03390504	A Study of Erdafitinib compared with Vinflunine or Docetaxel or Pembrolizumab in participants with advanced urothelial cancer and selected Fibroblast Growth Factor Receptor (*FGFR*) gene aberrations	Urothelial Cancer	**Erdafitinib**, Vinflunine, Docetaxel, Pembrolizumab	631
NCT04197986	Study of oral Infigratinib for the adjuvant Treatment of subjects with invasive urothelial carcinoma with susceptible *FGFR3* genetic alterations	Upper Tract Urothelial Carcinomas, Urothelial Bladder Cancer	**Infigratinib**	218
NCT04093362	Futibatinib versus Gemcitabine-Cisplatin chemotherapy as first-line treatment of patients with advanced cholangiocarcinoma harboring *FGFR2* gene rearrangements	Advanced Cholangiocarcinoma; *FGFR2* Gene Rearrangements	**Futibatinib**, Cisplatin/Gemcitabine	216
NCT03773302	Phase 3 study of BGJ398 (Oral Infigratinib) in first line cholangiocarcinoma with *FGFR2* gene fusions/translocations	Advanced Cholangiocarcinoma, *FGFR2* Gene Mutation	**Infigratinib**, Gemcitabine, Cisplatin	300
NCT03656536	A study to evaluate the efficacy and safety of Pemigatinib versus chemotherapy in unresectable or metastatic cholangiocarcinoma	Unresectable Cholangiocarcinoma, Metastatic Cholangiocarcinoma	**Pemigatinib**, Gemcitabine, Cisplatin	432
NCT03784014	Molecular profiling of advanced soft-tissue sarcomas	Soft Tissue Sarcoma	Nilotinib, Ceritinib, Capmatinib, Lapatinib, Trametinib, Combination of Trametinib and Dabrafenib, Combination of Olaparib and Durvalumab, Palbociclib, **Futibatinib**	960
